# Species diversity and molecular analysis of opportunistic *Mycobacterium, Nocardia and Rhodococcus* isolated from the hospital environment in a developing country, a potential resources for nosocomial infection

**DOI:** 10.1186/s41021-021-00173-7

**Published:** 2021-01-28

**Authors:** Marzieh Siavashifar, Fatemeh Rezaei, Tahereh Motallebirad, Davood Azadi, Abdorrahim Absalan, Zahra Naserramezani, Mohadeseh Golshani, Morteza Jafarinia, Kazem Ghaffari

**Affiliations:** 1 Student Research Comitee, Khomein University of Medical Sciences, Khomein, Iran; 2 Department of Basic and Laboratory and Sciences, Khomein University of Medical Sciences, Qods street, Khomein, Iran; 3 Department of Immunology, School of Medicine, Isfahan University Of Medical Sciences, Isfahan, Iran

**Keywords:** 16S rRNA, Actinomycetes, Hospital environment, Nosocomial infection

## Abstract

**Background:**

Hospital environmental resources have a significant role in cross-transmission of opportunistic pathogens such as actinomycetes species to the patients. Actinomycetes have a remarkable capability to survive in adverse and harsh conditions of hospital environments; therefore, they are a threat to the health of patients. Due to this issue, we aimed to determine the frequency and diversity of actinomycetes species in hospital soil, water and dust by using a combination of conventional and molecular methods including the phenotypic and biochemical tests for preliminary identification and the PCR amplification of the specific region of the 16S rRNA, *hsp*65 gene and sequence analyses of 16S rRNA for the genus and species identification.

**Results:**

A total of 50 (35.2%) actinomycetes isolates from 7 genera were isolated from 142 hospital environmental samples. The three most prevalent species were *M. setense* 10%, *R. erythropolis* and *M. fortuitum* 8% followed by *N.cyriacigeorgica* and *M. gordonae* 6%, *M. chelonae, M. abscessus, M. lentiflavum, M. mucogenicum, N. asteroides, N. farcinica, R. equi* and *L. shinushuensis* 4% and the single isolates of *M. conceptionense, M. septicum, N. rhamnosophilia, N. bravicatena, M. flavescens, M. arupense, M. doricum, M. frederiksbergense, S. heliomycini, S. albus, S. albogriseolus, R. facians, D. maris, G. terae* and *A. globiformis.*

**Conclusions:**

In conclusion we showed that the hospital environment is a potential reservoir for a broad range of actinomycetes species, due to the remarkable survival capability of these microorganisms in adverse hospital environment, carrying a threat to the health of patients.

## Introduction

Actinomycetes is a general term for the heterogeneous group of gram-positive bacteria with fungal morphology growing as anaerobic facultative or aerobic rods [1]. They are widely distributed in nature, particularly in soil and water and they make a considerable portion of the soil microflora (10^+ 4^10^+ 6^ CFU/mL) (10^+7^W22; 10^+ 8^ CFU/mL) and also found as members of the normal microbiota in open cavities, upper respiratory tract, especially the oropharynx, female genital tract, and gastrointestinal tract [2, 3].

Bacteria in the actinomycetes group consist of more than eight genera and 500 officially recognized species [4]. The major genera in the actinomycetes group are *Actinomyces*, *Corynebacterium, Mycobacterium*, nocardioform bacteria, and *Streptomyces*. Among the different identified actinomycetes species, only a few of them such as *Nocardia, Mycobacterium*, and Actinomyces cause infection in human and animal. However, nowadays several other species are being increasingly recognized as significant pathogens in immunocompromised patients such as patients with AIDS, transplanted neoplastic diseases, diabetic patients, patients under immunosuppressive therapy, and those with autoimmune disorders and cancerous patients [5–7]. However, several recent reports indicated that bacteria belonged to actinomyces group can produce infections in immune-competent population with no preexisting illness, trauma, or immunosuppressive therapy [8, 9]. Some of these infection due to actinomycetes group are as follows: *N. cyriacigeorgica, N. beijingensis* and *N. asteroides* caused brain and organ abscess, *M. avium and M. smegmatis* caused pulmonary infection, *A. meyeri* and *R. equi* caused disseminated infection [10–13].

The ubiquitous nature of actinomycetes allows their persistence in environmental biofilms. This capability within hospitals environments such as medical devices and water pipes or other healthcare units represents a threat to human health are present in hospital. This issue caused colonization, pseudo-infection or infection during hospitalization or after discharge by more-indolent organisms, such as actinomycetes species [14, 15]. Due to increased incidence of diseases and nosocomial infections in immune-competent population with actinomycetes group underlined the important effects of actinomycetes genera on human health [15–17].

There is no evidence for person-to-person transmission of actinomycetes, nevertheless the high-resistance nature of actinomycetes species allows their persistence in adverse and harsh conditions such as hospital environment [18]. This survival capability within hospital environments such as water resources, soil and dust and medical devices represents a threat to health of susceptible hosts including patients and hospital staff. They respond to variations such as heat or cold shocks, oxygen deprivation, pH changes, exposure to toxins and antibiotics in the environment by exhibiting altered growth and characteristics that favor the onset and spread of hospital-acquired infection (HAI) [19].

Although many of the opportunistic infections associated with actinomycetes have been reported to be common in developed countries such as Iran [20]. However, there has been a few extensive reports on the diversity and potential inhabitants of actinomycetes in hospital environments from developing countries [14, 21].

The identification of actinomycetes species in the developing countries has been done using conventional microbiological methods such as acid-fast staining, semi-quantitative, and heat-stable (68 °C) catalase production and niacin accumulation, pyrazinamide and growth on MacConkey agar without crystal violet [22], though isolation and accurate identification of isolates are difficult. Molecular tests that are currently applied provide a conclusive approach for the identification of Nontuberculous Mycobacteria (NTM). The molecular test strategies most often used are PCR-based RFLP (PRA) and sequence analysis of variable regions within microbial conserved genes, including *rpo*B or *hsp*65 and 16S rRNA [23, 24].

The aim of this study was to appraise the frequency and diversity of actinomycetes species which have the survival capability in the hospital environments, by applying molecular and phenotypic microbiologic methods in order to provide a better insight into their likely role as a reservoir for the transmission and development of nosocomial infections.

## Materials and methods

### Sampling

For isolation of environmental actinomycetes from July 2018 to November 2019, a total number of 142 environmental samples including dust, soil and drinking and non-drinking water (tap water, shower and well water) were collected aseptically in sterile bottles from different departments and environments of 13 hospitals in the Markazi province of Iran. Temperature and pH for each sample were measured by standard methods. The samples were processed based on standard protocols.

In summary, for aquatic samples, each liter was transported at 4 °C to the laboratory and processed within a maximum period of 24 h. Since the final goal was to screen and isolate mycobacterial species, initially the collected water samples were treated with 0.005% cetylpyridinium chloride (CPC) for 15 min to reduce the number of not-desirable microbial contaminants such as fungi, protista and other bacteria. Afterwards, the pretreated samples were subjected to vacuum filtration (cellulose nitrate 0.45 μm, Sartorius AG, Gottingen, Germany). The filters were rinsed and soaked in tubes containing 15 mL of distilled water. Almost 100 mL aliquots of dissolved filters were transferred into tubes of Lowenstein-Jensen (LJ) media and sauton’s media supplemented with antifungal and antibacterial antibiotics [kanamycin, nystatin and nalidixic acid (each at 50 μg mL^− 1^)], and was incubated at a temperature between 25 °C, 35 °C and 42 °C in an atmosphere of 5% CO2 for 8 weeks [25].

For soil samples, 3–5 g soil were taken from potentially microbial contamination site in the hospital environment include: hospital green space, pots inside the patient’s room, door and window labels, patient beds, hospital equipment, and nursing station. Then transferred directly to the laboratory. Three grams of soil were transferred to 50 mL sterile centrifuge tube. Then, 20 mL sterile distilled water was added to the tube and shacked for 5 min, and centrifuged at 4300 RCF at room temperature for 20 min. The pellet and supernatant were decontaminated in separate tubes using 3% sodium lauryl sulfate and 1% NaOH (Kamala et al., 1994a). Afterwards, 100 μL of the decontaminated sample was used to inoculate LJ media and sauton’s media and was incubated at 25°C, 35°C and 42 °C with 5% CO_2_ atmosphere for 8 weeks [26].

For the dust samples, 2–3 g dust were collected from the floors and window panes of patients’ rooms with a sterile swab or brush, suspended in 100 mL distilled water for 60 min, and allowed to stand at room temperature for additional 10 min. Tenfold dilutions of the homogenized suspensions were prepared and 100 μl of each of the pretreated 10^− 2^, 10^− 3^, and 10^− 4^ dilutions were inoculated into LJ media and Sauton’s media and incubated for 8 weeks at 25°C, 35°C and 42 °C with 5% CO_2_ atmosphere [27]. The details of environmental samples tested during this study are given in Table 1.

**Table 1:** Samples profile and phenotypic and molecular features of actinomycetes hospital environmental isolates

	Isolates	Sample profile						Phenotypic features										16S rRNA analysis		
Number of isolates	Designation	Sample type	Hospital department	Tm	TDS for water samples	pH	Opt. Tm	Growth rate	Pigment production	Nitrate reduction	Catalase	Tween 80 hydrolyses	Urease	Lysozyme Resistance	Decomposition of Tyrosine	Decomposition of Xanthene	Decomposition of Hypoxanthine	Similarity (%)	Base pair differences	Identification
5	AE13/AE14/AE15/AE16/AE17	Soil/Drinking water	Men Surgery / Emergency	7–14	200	7.4	35	R	N	+	+	–	+	–	–	–	–	100	0/925	*M. setense*
2	AE20/AE21	Soil/Well water	Pediatrics / Maternity	12–21	582	7.6	35	S	N	–	–	–	**–**	–	–	–	–	100	0/830	*M. lentiflavum*
1	AE4	Shower water	Women Surgery	20	312	7.2–7.8	35	S	N	–	+	–	**+**	–	–	–	–	99.9	1/640	*M. conceptionense*
2	AE2/ AE3	Dust /Soil	ICU/ Oncology	20–26		8	35	R	White	+	–	+	**–**	–	–	–	–	100	0/735	*M. mucogenicum*
1	AE26	Drinking water	General	18	192	7.4	30	R	White	+	–	+	**–**	–	–	–	–	100	0/870	*M. septicum*
4	AE5/AE6/AE7/AE8	Dust/Soil/ Drinking Water	Men Surgery/ Maternity	14–21	265	7–8.2	35	R	N	+	–	–	–	–	–	–	–	100	0/959	*M. fortuitum*
2	AE18/ AE19	Soil	Emergency/ ICU	22–26		6.9	35	S	N	+	–	+	–	–	–	–	–	100	0/959	*M. chelonae*.
3	AE23/AE24/AE25	Drinking Water/ Shower water	Neurosurgery/ Ophthalmology	20–30	168–245	7.2–8	35	S	Orange	+	+	–	–	–	–	–	–	100	0/860	*M. gordonae*
1	AE42	Soil	Women Surgery	33		7.6	35	S	Pinkish	+	–	–	–	+	–	–	–	100	0/790	*N. rhamnosophilia*
1	AE39	Soil	Pediatrics	30		7.6	30	R		–	+	–	–	+	–	–	–	100	0/830	*N. bravicatena*
1	AE9	Dust	Emergency	9		7.3	30	R	Yellow	+	+	–	+	–	–	–	–	100	0/830	*M. flavescens*
2	AE11/AE12	Soil/Drinking water	Ophthalmology	8–32	96	6.7–8	35	R	N	+	+	–	+	–	–	–	–	99.9	1/977	*M. massiliense/abscessus* complex
1	AE22	Well water	Green space	13	412	7	35	R	N	–	+	–	–	–	–	–	–	99.9	1/880	*M. arupense*
2	AE40/AE41	Soil/Shower water	Emergency/ Oncology	12–22	212	8.2	35	R	Pink	–	–	+	+	+	–	–	–	100	0/847	*N. asteroides*
2	AE34/AE35	Dust	Men Surgery/ICU	8–21		7.8	35	R	Orange	–	+	–	+	+	–	–	–	99.78	2/800	*N. farcinica*
1	AE10	Water	Infectious diseases	25		8	30	S			–	+	–	–	–	–	–	100	0/920	*M. doricum*
3	AE36/AE37/AE38	Soil/Drinking water	Men surgery/ Infectious disease	8–16	156	6.6–7.4	35	R	White	+	+	–	–	+	–	–	–	100	0/912	*N. cyriacigeorgica*
1	AE1	Soil	Emergency	13		8.2	35	R	Yellow	–	+	+	–	–	–	–	–	99.9	1/932	*M. frederiksbergense*
1	AE43	Soil	Pediatrics	13		7.1	25	R	Yellow	+	+	–	+	–	+	+	+	100	0/694	*S. heliomycini*
1	AE44	Soil	Emergency	16		8	30	R	Red	–	+	–	+	–	–	–	+	100	0/763	*S. albus*
1	AE45	Dust	Surgery	21		7.8	30	R	White	+	+	–	+	–	+	+	–	100	0/940	*S. albogriseolus*
4	AE27/AE28/AE29/AE30	Soil/Drinking water	Men Surgery / Emergency	14–22	112	6.3	30	R	White	+	+	+	+	–	+	+	–	100	0/853	*R. erythropolis*
2	AE31/AE32	Soil/Shower water	Pediatrics / Maternity	28	195	6.8	30	R	Yellow	+	+		+	–	+	+	+	99.9	2/993	*R. equi*
1	AE33	Well Water	Women Surgery	21	523	7.2	30	R	N	–	+	–	+	–	–	–	+	100	0/685	*R. facians*
1	AE46	Soil	Infectious diseases	13		7.3	30	R	White	–	+	–	+	–	–	+	–	100	0/745	*A. globiformis*
2	AE47/AE48	Drinking Water	Men surgery/ Infectious disease	16	143	6.7–8	30	R	Reddish	+	+	–	–	–	–	–	–	100	0/960	*L. shinushuensis*
1	AE48	Dust	Emergency	21		7	30	R	White	+	–	+	–	–	–	–	–	100	0/800	*G. terae*
1	AE49	Soil	Pediatrics	14		8.2	30	R	White	+	+	–	–	+	–	–	–	100	0/850	*D. maris*

1; Tm; Temperature

2; R; Rapid grower < 7 days; S: Slow grower > 7 days

3; Similarity; % similarity to the nearest validated species

4; Base pair differences: the number of nucleotide differences between isolates and the nearest validated species

5. TDS: total dissolved solids

This study was approved by the ethics committee Khomein University of Medical Sciences (Grant No. 3021).

### Conventional identification of isolates

The environmental isolates were characterized phenotypically as actinomycetes group (nocardia, rhodococcus, mycobacterium, streptomyces, actinomyces, gordonae and arthrobacter) applying conventional phenotypic and biochemical tests. The tests included acid-fast and partial acid- fast staining, tween opacity, semi-quantitative, and heat-stable (68 °C) catalase production, growth at 25, 32, 37, and 42 °C, urease activity, niacin accumulation, nitrate reduction, resistance to lysozyme, hydrolysis of tyrosine, xanthine, hypoxanthine tests tellurite reduction, and pigment production [18, 28].

### Molecular identification

#### DNA extraction and purification

Chromosomal DNA was extracted using simple boiling method or pitcher method with some modifications, [29, 30]. In modified pitcher method, lysis of actinomycetes cells carried out using sonication pretreatment followed by cell wall disruption with lysozyme (200 mg/mL final concentration) and proteinase K (300 μg/mL final concentration) in the presence of sodium dodecyl sulfate (SDS) and finally treatment with guanidine-sarcosyl solution. After extraction of the DNA-containing aqueous phase with phenol-chloroform-isoamyl alcohol (25:24:1, vol/vol/vol) and chloroform-isoamyl alcohol (24:1, vol/vol), DNA was precipitated with ethanol at − 20 °C. Precipitated DNA was washed with 70% ethanol and re-suspended in 100 μl of Milli-Q water (23). Simple boiling method was performed as follow: a few colonies of bacteria were added into 200 mL of TE buffer (Tris EDTA), boiled for 30 min and centrifuged at 10000 rpm for 10 min. The supernatant was transferred to another sterile microtube and centrifuged at 13000 rpm for 10 min. Precipitated DNA was re-suspended in 50 μl of Milli-Q water and stored at − 20 °C (24).

#### Molecular identification of actinomycetes isolates to genus and species level

The environmental isolates identified phenotypically as actinomycetes were further analyzed for genus and species levels using a panel of molecular tests including a genus-specific PCR based on a 618-bp fragment of the 65-kDa heat shock protein for NTM as recommended by Khan and Yadav [31] and, a 596 bp region of the 16S rRNA for streptomyces, rhodococcus and nocardia as recommended by Laurent [32], followed by the amplification and direct analysis of almost complete 16S rRNA sequencing for species identification as described by Shojaei et al. [9]. Sequencing was performed by ABI 3100 genetic analyzer in the Bioneer Company (South Korea), and the sequence data received were aligned manually with existing sequences of actinomycetes retrieved from the GenBank database and analyzed using the Blast program in GenBank and the jPhydit program [33].

#### Nucleotide sequence accession numbers

The GenBank accession numbers for the 16S rRNA sequencing of isolated actinomycetes in this study are listed below.*, M. mucogenicum* (MT705619)*, M. conceptionense* (MT705620), *M. abscessus* (MT705885), *M. frederiksbergense* like (MT708075)*, M. doricum* (MT708129)*, M. flavescens* (MT708131)*, M. fortuitum* (MT708130)*, M. septicum* (MT708132), *M. setense* (MT708076), *M. lentiflavum (*MT708077) and *M. gordonae* (MT705886), *R. equi* (MT734027), *R. facians* (MT734027), *R. erythropolis* (MT734047), *N. farcinica* (MT734045), *N. cyriacigeorgica* (MT734049), *N. bravicatena* (MT734050), *N. asteroides* (MT734070), *N. rhamnosophila* (MT734071), *S. albus* (MT734111), *S. albogriseolus* (MT734112), *A. globiformis* (MT734564), *L. shinushuensis* (MT734570), *D. maris* (MT734648) .

## Result

**Fig. 1:**
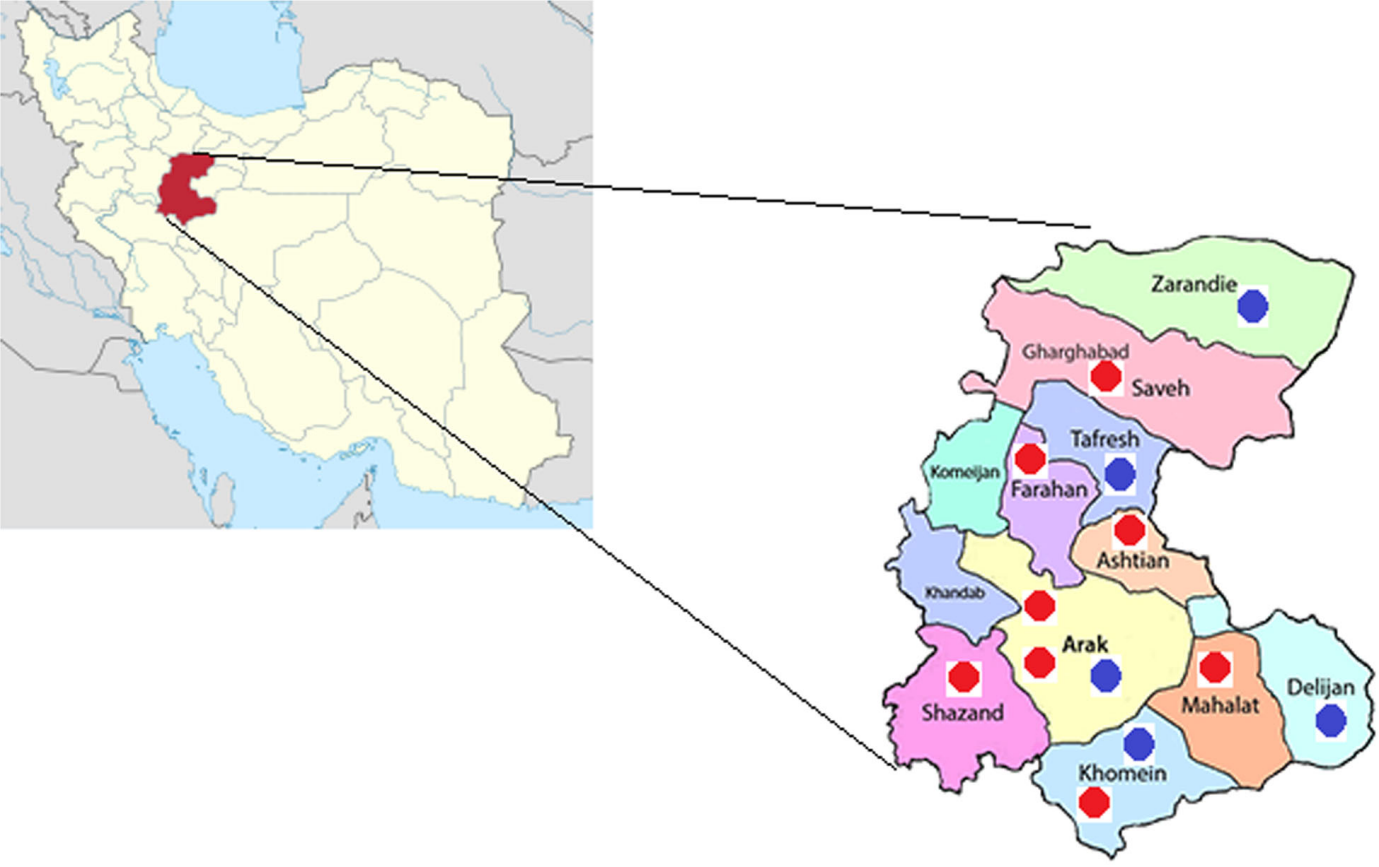
Geographic distribution of sampling site from markazi province hospitals, blue spots are negative and Red spot are positive samples for isolation of actinomycetes. The Figure source is from Iran National mapping agency and designed by Adobe Photoshop 2020 v21.2.2.289

In this study, a total of 50 (35.2%) actinomycetes isolates were collected from 142 water, soil and dust samples collected from Markazi province hospitals. Total of 28 (19.7%) samples were contaminated with gram-positive and gram-negative bacteria and fungi. The positive samples came from the 8 of 13 hospitals; no actinomycetes were detected in any of the analyzed samples taken from the 5 other hospitals (Fig. 1). Of the total isolates, 32 isolates were recovered from dust and soil samples and 18 isolates were recovered from water samples.

The recorded temperature and the pH of the water samples were between 9 and 33 °C and 6–8.5, respectively. The total dissolved solids for the water samples ranged between 284 and 618 mg/L. The corresponding figures for soil samples were in the range of 11 to 28 °C 6.8 to 8.5. The details of dust, soil and water samples and the isolates are shown in Table 1.

On the basis of morphological, culture, and biochemical features, and the genus specific markers such as, presence of a 618-bp fragment of the hsp65 and a 596 bp fragment of the 16S rRNA, all 50 isolates were identified as actinomycetes (*Nocardia, Rhodococcus, Arthrobacter, Streptomyces, Leifsonia, Dietzia, and Mycobacterium*), of which 26 isolates were identified as mycobacterium, nine isolates were identified as nocardia, three isolates were identified as streptomyces, two isolates were identified as leifsonia and one isolate was identified as arthrobacter and dietzia, of each.

16S rRNA gene sequences’ analysis of the isolates revealed that all isolates had nucleotide signatures of actinomycetes, at positions 70–98 (U-A), 139–224 (G-C), 843(C), 1008–1021 (C-G), 1189 (C), 1244–129 (C-G) and 1308–1329 (C-G) for mycobacterium and at positions 70–98 (A-T), 293–304 (G-T), 307 (C), 328 (T), 614–626 (A-T), 631(G), 661–744 (G-C), 824e876 (T-A), 825–875 (A-T), 843 (C), and 1122–1151 (A-T) for nocardia and streptomyces [34].

The most prevalent Iranian actinomycetes species isolated from hospital environmental samples were *M. setense* 5 isolates (10%), *M. fortuitum* and *R. erythropolis 4* isolates each (8%), *N.cyriacigeorgica and M. gordonae* 3 isolates each (6%), *M. chelonae, M. abscessus, M. lentiflavum, M. mucogenicum, N. asteroides, N. farcinica, R. equi* and *L. shinushuensis* 2 isolates each (4%) and 15 species including., *M. conceptionense, M. septicum, N. rhamnosophilia, N. bravicatena, M. flavescens, M. arupense, M. doricum, M. frederiksbergense, S. heliomycini, S. albus, S. albogriseolus, R. facians, D. maris, G. terae* and *A. globiformis* consisted a single isolate. The almost complete 16S rRNA gene sequences obtained from the species’ accurate identification of clinical isolates represented that all isolates had 100% similarity with the nearest standard type strain of actinomycetes species.

**Fig. 2:**
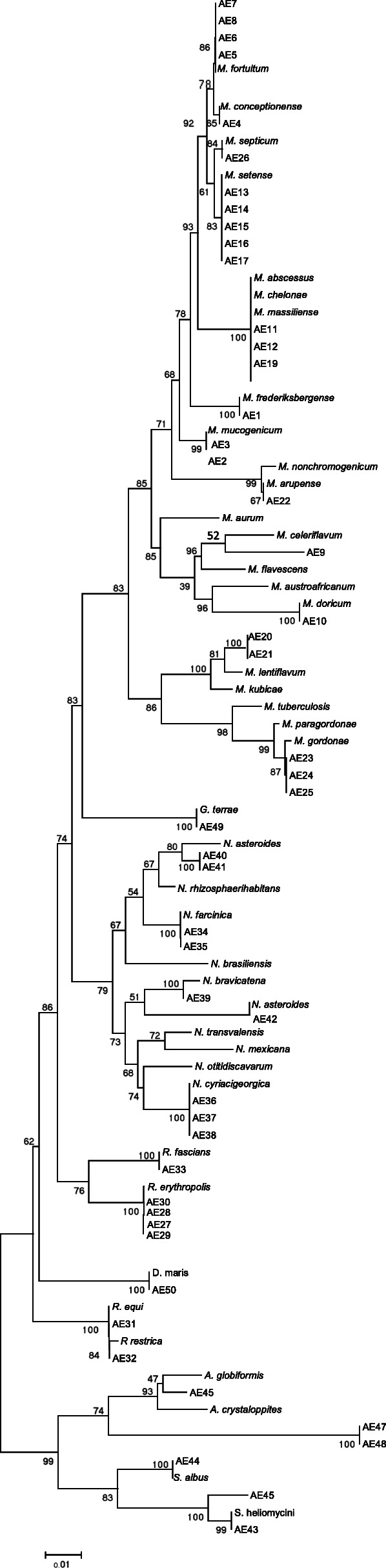
16S rRNA sequence based phylogenetic tree for environmental actionmycetes and nearest validated species of actinomycetes by using the neighbor-joining method. The figures at each node represent bootstrapping values

The phylogenic relationship between environmental actinomycetes isolates and the validated actinomycetes species was presented by phylogenetic tree of 16S rRNA and by the high bootstrap value obtained using the neighbor-joining method with arithmetic mean using the matrix of pairwise differences. (Fig. 2).

## Discussion

Members of actinomycetes group are widely distributed in nature and are found in water, dust, soil, and decaying vegetation [18], and also frequently found as members of the normal microbiota of oropharynx, gastrointestinal tract, upper respiratory tract, and female genital tract of human and animal in [35].

Nowadays infections due to different species of actinomycetes such as nocardia, mycobacterium, rhodococcus, streptomyces, gordonae and actinomyces. Have gained considerable importance because of their increasing incidence in immunocompromised patients as well as in immunocompetent patients [36, 37]. Infections due to actinomycetes group members are clinically hard to differentiate from *M. tuberculosis* [38, 39]. Some actinomycetes species based on their high lipid content and triple-layered cell wall, they have a remarkable stress tolerance, which leads to more resistant to killing in a harsh environmental condition such as normal disinfection, elevated temperature, and ultraviolet light compared with other pathogenic bacteria that may colonize in hospital environment [40]. Due to this issue the use of colonized aqueous solutions and inadequate sterilization or disinfection of hospital room and environment and equipment are often factors in nosocomial infections by actinomycetes species. Despite of these group’s ability to spread by aerosolization and direct contact to human and their slow growth, there is a significant reason to study actinomycetes species in various environments and that is the fact that these species may play the role of emerging pathogens [41, 42].

Water, soil and decaying vegetation are the major sources of these opportunistic bacteria [43]. Nevertheless, environmental resources of hospitals, especially in developing countries are not evaluated for the presence of actinomycetes group species. On the other hand, the majority of bacteriological tests commonly used in laboratories are not able to detect actinomycetes species in water, soil and dust [44, 45]. This may be the most important but neglected factor influencing the spread of these opportunistic organisms in critical environments, including nursing homes, hospitals, prisons and other similar places [46, 47]. This fact is described by reports of cases such as gastrointestinal tract infections, ocular infections, pulmonary infections and cutaneous or soft tissue infections particularly in post-surgery patients [48–50].

Likewise, contamination of hospital medication and equipment, traced for the persistence of actinomycetes species in hospital environmental resources and their resistance to commonly used disinfectants [51], was responsible for pseudo-outbreaks of infections related to surgical implants, lung disease following bronchoscopy and health care-associated septicemia [48, 50].

Increasing isolation of actinomycetes species from environmental and clinical sources in different parts of the world [52–55] prompted us to design the present study to determine the extent of the actinomycetes diversity in hospital environmental samples of Markazi province of Iran. We envisaged that optimization of methods in isolation and accurate identification of actinomycetes group species recovered from water, soil and dust samples by application of conventional microbiologic and molecular approaches is a key step towards the description of the emerging infections that they cause but are often ignored or missed.

In the current study, our isolation rate of actinomycetes species from hospital environmental resources samples was found to be 35.2%. We were unable to compare the isolation rate of actinomycetes species in this study with that of other studies, since a thorough literature review revealed there were no published research studies on isolation of actinomycetes species from water, dust and soil samples in hospitals.

The actinomycetes species recovered from environmental samples in our study were 13 mycobacterial species included *M. fortuitum, M. chelonae, M. abscessus, M. lentiflavum, M. mucogenicum, M. setense, M. conceptionense, M. septicum, M. gordonae, M. flavescens, M. arupense, M. doricum,* and *M. frederiksbergense,* and four nocardia species included *N. rhamnosophilia, N. bravicatena, N. asteroides,* and *N. farcinica*, three rhodococcus and streptomyces species included, *R. equi. R. facians, R. erythropolis, S. albus, S. albogriseolus,* and *S. heliomycini* and one species of *D. maris, G. terae, A. globiformis* and*, L. shinushuensis*. Based on literature of review, among the actinomycetes species isolated in this study, 16 species and 11 species were shown that are opportunistic pathogens and non- pathogens, respectively. In our study, some similar actinomycetes strains were isolated by sampling from different places of a hospital ward, which indicated that the entire ward was polluted with these strains.

Result of our study showed that *M. setense*, was the most encountered actinomycetes included 5 isolates (10%), following that *M. fortuitum, and R. erythropolis* included 4 isolates of each (8%). These findings are in accordance with another report from various parts of the world showing *M. fortuitum, R. erythropolis and M. setense* are most common actinomycetes species isolated from environmental sample [56–59]. In the current study these species were isolated from soil dust and water samples of hospitals.

*N. cyriacigeorgica and M. gordonae* ranked third included 6% (3 Isolates) of all the isolates. *N. cyriacigeorgica is* a human opportunistic pathogen that was first isolated and characterized, in 2001, from the bronchial secretions of a patient with chronic bronchitis [60] and *M. gordonae* is one of the most common mycobacterial species was isolated from hospital water resources [61]. In this study we isolated this species from water and the soil of decayed vegetation pots in patient’s room in hospitals.

In our study, eight isolates included *M. chelonae, M. abscessus, M. lentiflavum, M. mucogenicum, N. asteroides, N. farcinica, R. equi* and *L. shinushuensis* ranked fourth with a frequency of 2 (4%) isolates each among all Iranian actinomycetes isolates. *M. lentiflavum* is a rare slow-growing human opportunistic pathogen closely related to *M. simiae* and *M. genavense* [62, 63]. *M. mucogenicum* is a rapidly growing mycobacterium found ubiquitously in water resources. It has been reported as a cause of widespread infections entering from wound or central venous catheters especially in immunocompromised patients [64]. In this study these species were isolated from water and soil samples in hospitals.

*M. chelonae and M. massiliense/abscessus* complex is a rapidly growing mycobacterium that is a common water contaminant [65]. Several reports have indicated that these organisms can be the cause of post-traumatic wound infections, chronic lung disease, ocular infection, disseminated cutaneous diseases and persistent culture-negative skin infections, mostly in patients with suppressed immune system, although *M. chelonae* can cause the pseudo-epidemics in hospitals [66, 67]. In this study these species were isolated from water samples in hospitals.

*N. asteroides* and *N. farcinica* are the first historically identified nocardia species and have been the most commonly reported isolates from clinical and environmental samples worldwide [4]. *R. equi, R. facians* and *L. shinushuensis* generally found in dry and dusty soil and water resources and can be important for diseases of domesticated animals [4]. In this study we recovered these species from water, distilled water, dust and soil samples in hospitals.

In the current study we reported isolation of *M. conceptionense, M. septicum, N. rhamnosophilia, N. bravicatena, M. flavescens, M. arupense,* and *M. doricum,* taken from hospital water and dust samples. These organisms are rare actinomycetes originally isolated from clinical specimens; our study reports the isolation of these organisms from the hospital environmental resources such as water, soil and dust [68–70]. This could be an indicative of the source of these microorganisms in the environment.

*M. frederiksbergense S. heliomycini, S. albus, S. ablogriseolus, D. maris, G. terae* and *A. globiformis* were commonly found in soil and sediment, and have the biodegrading ability of pollution, as well as produced antimicrobial agent [71, 72]. To date, there have been no reports on the clinical significance of these organisms [72].

## Conclusion

In conclusion, the present study showed that hospital environmental resources can be the reservoir of a wide range of opportunistic actinomycetes which can act as a potential supplier for transmission of opportunistic infections to patients. Moreover, our study once more signified the fact that the occurrence of actinomycetes species in hospital environment is worth to be considered, since these organisms show a high degree of intrinsic resistance to common antiseptic and disinfectant solutions and are able to threat patients’ health, particularly in those suffering from weakness of immunity. Furthermore, taking the role of a variety of hospital devices in transmission of actinomycetes to vulnerable patients into consideration might be of interest and may help to the better assessment of problem dimensions.

## Data Availability

The data that support the findings of this study are available from the corresponding author upon reasonable request.

## Abbreviations

AIDS: Acquired immunodeficiency syndrome
HAI: hospital-acquired infection
NTM: Nontuberculous Mycobacteria
PRA: PCR-based RFLP
CPC: cetylpyridinium chloride
LJ: Lowenstein-Jensen
SDS: sodium dodecyl sulfate
TE: Tris EDTA
