# Correction to: Species diversity and molecular analysis of opportunistic Mycobacterium, Nocardia and Rhodococcus isolated from the hospital environment in a developing country, a potential resources for nosocomial infection

**DOI:** 10.1186/s41021-021-00178-2

**Published:** 2021-03-05

**Authors:** Marzieh Siavashifar, Fatemeh Rezaei, Tahereh Motallebirad, Davood Azadi, Abdorrahim Absalan, Zahra Naserramezani, Mohadeseh Golshani, Morteza Jafarinia, Kazem Ghaffari

**Affiliations:** 1 Student Research Comitee, Khomein University of Medical Sciences, Khomein, Iran; 2 Department of Basic and Laboratory and Sciences, Khomein University of Medical Sciences, Qods street, Khomein, Iran; 3 Department of Immunology, School of Medicine, Isfahan University Of Medical Sciences, Isfahan, Iran

**Correction to: Genes and Environ 43, 2 (2021)**

**https://doi.org/10.1186/s41021-021-00173-7**

In the original publication [1] the wrong affiliation was shown for Dr. Ghaffari, the original publication stated the following:

- 1 Student Research Comitee, Khomein University of Medical Sciences, Khomein, Iran.

However, the correct affiliation is:

- 2 Department of Basic and Laboratory and Sciences, Khomein University of Medical Sciences, Qods street, Khomein, Iran.

The original article has been updated.
